# Midbrain Ischemic Strokes Presenting as Isolated Internuclear Opthalmoplegia

**DOI:** 10.7759/cureus.17819

**Published:** 2021-09-08

**Authors:** Dalys Haymes, Matthew Jenson, Chidi Nwachukwu, Peter Fiester

**Affiliations:** 1 Neuroradiology, University of Florida College of Medicine – Jacksonville, Jacksonville, USA; 2 Radiology, University of Florida College of Medicine – Jacksonville, Jacksonville, USA

**Keywords:** stroke, midbrain, vascular supply, infarct, internuclear ophthalmoparesis

## Abstract

Stroke can present with unique neurologic symptoms, which can be used to help determine the location of the stroke. Internuclear ophthalmoparesis (INO), also known as internuclearophthalmoplegia, is a distinct gaze abnormality with impaired horizontal eye movements with compromised adduction of the affected eye, and abduction nystagmus of the contralateral eye. Infarcts involving the medial longitudinal fasciculus in either the pons or midbrain can result in INO. We present two cases of midbrain ischemic stroke, which presented as isolated INO. The midbrain has a unique and intricate vascular supply including branches from the basilar, superior cerebellar, posterior cerebral, posterior communicating, anterior choroidal, and posterior choroidal arteries, which is reviewed. Infarcts involving the paramedian midbrain, which is supplied by short circumferential arteries and penetrating branches arising from the posterior cerebral artery and superior cerebellar artery, can result in INO.

## Introduction

Internuclear ophthalmoparesis (INO), also referred to as internuclearophthalmoplegia, is a distinct gaze abnormality characterized by impaired horizontal eye movements, compromised adduction of the affected eye, and abduction nystagmus of the contralateral eye. The underlying pathology most commonly encompasses disruption of the medial longitudinal fasciculus (MLF) of the side displaying inadequate adduction, which is usually the result of a pontine infarct [1]. Midbrain lesions may also result in disruption of MLF fibers as they project to the contralateral oculomotor nucleus upon traversing the dorsomedial tegmentum of the pons or midbrain [1].

Isolated unilateral internuclear ophthalmoplegia is a relatively rare occurrence and can be seen in a minor acute ischemic stroke involving the MLF. The midbrain exhibits a unique and intricate blood supply, which can lead to specific patterns of infarct depending on the affected vessel. We present two cases of isolated INO with MRI findings confirming stroke involving the MLF at the level of the rostral to middle midbrain.

## Case presentation

Case 1

**Figure 1:**
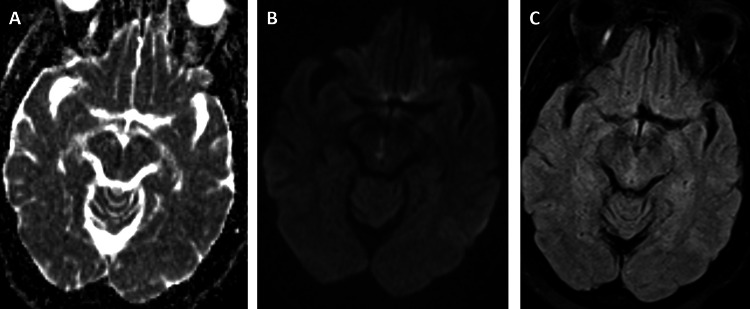
Case 1 MRI Axial MRI images including (A) ADC, (B) DWI, and (C) FLAIR.  Images demonstrate restricted diffusion with associated FLAIR signal abnormality in the right paramedian midbrain. ADC, apparent diffusion coefficient; DWI, diffusion-weighted imaging; FLAIR, fluid-attenuated inversion recovery.

A 52-year-old female with a history of hypertension, obesity, hyperlipidemia, and coronary atherosclerotic disease presented to the emergency department with inferior wall myocardial infarction . Following left heart catheterization, the patient developed dizziness in the setting of abnormal eye movement characterized by left INO. An MRI performed the same day (Figure 1) demonstrated infarcts of likely embolic etiology within the right midbrain along the course of the MLF. Additional embolic infarcts within the splenium of the corpus callosum and left caudate nucleus were also demonstrated upon that initial imaging. Craniocervical CT angiography demonstrated no vessel occlusion or stenosis, reflecting the likely cardioembolic origin in the setting of recent catheterization for intervention for myocardial infarction.

Case 2

**Figure 2:**
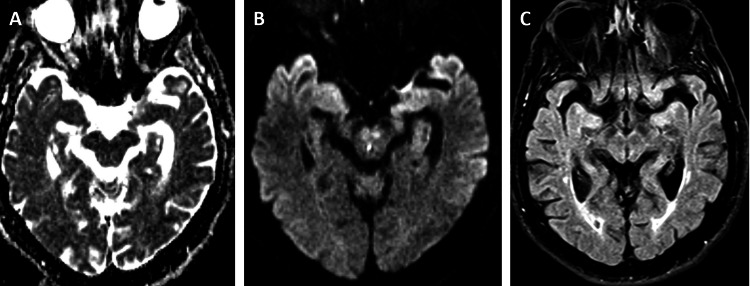
Case 2 MRI Axial MRI images including (A) ADC, (B) DWI, and (C) FLAIR.  Images demonstrate a small infarct in the right periaqueductal white matter along the anatomic course of the right MLF, without definite FLAIR correlate at this time. ADC, apparent diffusion coefficient; DWI, diffusion-weighted imaging; FLAIR, fluid-attenuated inversion recovery; MLF, medial longitudinal fasciculus.

A 72-year-old male with a history of hypertension, hyperlipidemia, chronic kidney disease, and chronic obstructive pulmonary disease presented to the ED with dizziness and visual changes consistent with right INO. MRI evaluation (Figure 2) revealed a small infarct in the right periaqueductal white matter along the anatomic course of the right MLF. Moderate periventricular white matter changes were also visualized and likely related to chronic small vessel ischemic changes. Craniocervical MR angiography evaluation demonstrated moderate stenosis in the bilateral carotid bulbs, greater on the left than right. No significant stenosis in the circle of willis was noted.

## Discussion

Isolated INO as presentation of focal midbrain infarcts is a very uncommon entity with few reported cases within the literature [2-4]. This fact is partially the result of the overlapping vascular supply of the midbrain with other structures, such as the thalamus [5]. The paramedian (PM) area infarction is most frequent, followed by lateral area infarction. Reported cases of midbrain stroke presenting as INO in the literature were found to have involved the PM region of the midbrain and most cases have been associated with diabetes mellitus [5] in addition to other medical diseases contributing to chronic arteriopathy [3,5]. This finding is consistent with previous reports of pure midbrain infarction. Nearly all cases result in visual disturbances that commonly contribute to perceptual symptoms, such as dizziness [3,5].

In contrary to what is typically described in the literature, our two patients have no history of diabetes mellitus; however, both patients have a history of hypertension, smoking, and hyperlipidemia, which are important risk factors for atherosclerosis. One case occurred immediately after cardiac catheterization with additional ischemic foci involving the splenium of the corpus callosum and caudate nucleus, consistent with cardioembolic phenomena, which is a rare cause of midbrain stroke. The other patient suffered small lacunar infarct likely related to microvascular atherosclerotic disease. Both patients developed dizziness, which may be related to involvement of the vestibular pathway in the region of the MLF.

**Figure 3:**
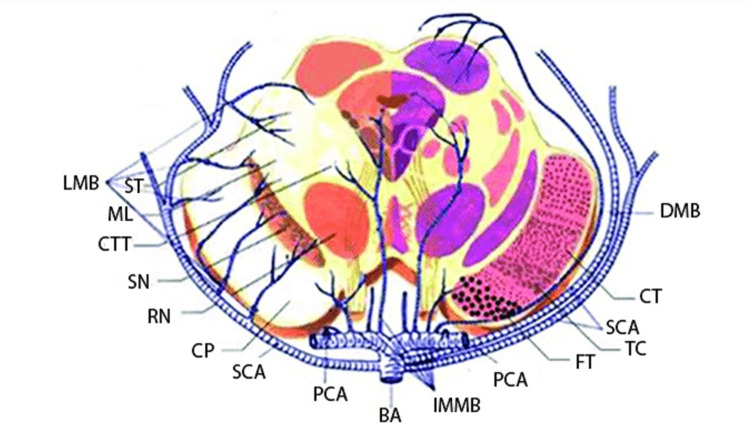
Vascular Supply of the Midbrain LMB, lateral mesencephalic branches; ST, spinoreticular tract; ML, medial lemniscus; CTT, central tegmental tract; SN, substantia nigra; RN, red nucleus; CP, cerebral peduncular; SCA, superior cerebellar artery; IMMB, inferior medial mesencephalic branch; FT, frontopontine tract; TC, tractus corticobulbrais; CT, corticospinal tract; DMB, dorsal mesencephalic branch. Image used with permission from the CC BY license from https://www.researchgate.net/publication/336800360_An_Analysis_of_Clinical_Characteristics_of_Rare_Bilateral_Cerebral_Peduncular_Infarction.

Our cases presented with small-volume infarcts located in the PM portion of the midbrain. The majority of the midbrain arterial supply is derived mostly from the superior cerebellar artery and the posterior cerebral artery and to a lesser degree from branches of the posterior communicating artery and posterior choroidal artery, all branches of the basilar artery (Figure 3). The anterior choroidal artery also supplies a portion of the midbrain.

The vascular supply of the midbrain is better understood by four distinct zones, which are the median, PM, posterior, and lateral zones. The median zone of the midbrain receives its supply from a vascular plexus in the interpeduncular fossa derived from perforating branches of the tip of the basilar artery, the posterior communicating artery, and up to six branches of the posterior cerebral arteries from the P1 and P2 segments [6]. This can be appreciated in 66% of individuals [7]. These branches are named the superior and inferior medial mesencephalic branches [8]. This system supplies the raphe region oculomotor complex, medial longitudinal fasiculus, red nucleus, substantia nigra, and crus cerebri. The superior medial mesencephalic branches (sMMB) have inner and outer branches. The inner branch runs between the red nuclei of both sides and approaches the oculomotor nerve nucleus and central gray substances around the cerebral aqueduct. The outer branch runs through the medial part of the substantia nigra and tegmental tract. The inferior MMB also consists of an inner branch that runs in the decussation of the superior cerebellar peduncle and central gray substance and an outer branch that runs through the medial part of the substantia nigra and tegmental tract.

The PM zone is supplied by short circumferential arteries and penetrating branches arising from the posterior cerebral artery and superior cerebellar arteries. One or more short circumflex branches arise from the P1 or less commonly P2 segments of the posterior cerebral arteries [6]. They supply the crus cerebri, substancia nigra, and midbrain tegmentum [7]. Branches that diverge from the proximal portion of the PCA are named the MMB [8]. Goto classified the MMB into the sMMB supplying the rostral part of the midbrain and the inferior MMB (iMMB) supplying the caudal part of the midbrain [8]. The sMMB consist of two branches. One branch runs between the red nuclei on both sides, and approaches the oculomotor nerve nucleus and central gray substances around the cerebral aqueduct (inner sMMB). The other branch runs at the medial part of the substantia nigra and the lateral part of the red nucleus, and approaches the central tegmental tract (outer sMMB). The iMMB also consist of two branches. One branch of iMMB runs in the decussation of the superior cerebellar peduncle, and approaches central gray substances (inner iMMB). The other branch runs through the medial part of the substantia nigra and tegmental tract (outer iMMB).

The posterior midbrain and lateral zones are supplied by long circumflex arteries. These are present in 96% of individuals [7]. Up to three long circumflex branches may be seen [6]. The posterior median zone corresponds to the superior colliculus, inferior colliculus and radicule, and decussation of the trochlear nerve and it is supplied by the long circumferential branches originating mainly from the posterior cerebral artery, penetrating branches of the superior cerebellar artery, and other penetrating branches from the posterior choroidal branch [6]. This artery is named the dorsal mesencephalic branch [8]. This tends to arise more distally than the short circumflex arteries, runs in the ambient cistern, and approaches the quadrigeminal plate [6]. When a prominent branch of the superior colliculi is present it may be called the collicular artery, which supplies the superior and inferior colliculus [6].

The lateral zone includes the cerebral peduncle excluding its medial part, substantia nigra, medial lemniscus, and reticular formation. This also receives long circumferential branches from the posterior cerebral arteries and penetrating branches from the superior cerebellar artery as that vessel courses around the midbrain [6]. Goto named these arteries lateral mesencephalic branches [8]. The lateral mesencephalic branches arise from the PCA at the rostral part of midbrain and from the SCA at the caudal part of the midbrain. In this series, PM area infarction was most frequent, followed by lateral area infarction. This finding is consistent with previous reports of pure midbrain infarction [9].

## Conclusions

INO is a distinct gaze abnormality characterized by impaired horizontal eye movements with compromised adduction of the affected eye, and abduction nystagmus of the contralateral eye. The underlying pathology most commonly encompasses disruption of the MLF often due to infarct. Understanding the connection between the associated clinical symptoms and underlying anatomy will help clinicians more accurately diagnose and better care for their patients.
